# Loss of RNA binding protein HuD facilitates the production of the senescence-associated secretory phenotype

**DOI:** 10.1038/s41419-022-04792-y

**Published:** 2022-04-11

**Authors:** Seungyeon Ryu, Myeongwoo Jung, Chongtae Kim, Hoin Kang, Sukyoung Han, Seongho Cha, Seung Min Jeong, Eun Kyung Lee

**Affiliations:** 1 Department of Biomedicine & Health Sciences, The Catholic University of Korea, Seoul, 06591 South Korea; 2 Department of Biochemistry, The Catholic University of Korea, Seoul, 06591 South Korea; 3 Catholic Institute for Visual Science, The Catholic University of Korea, Seoul, 06591 South Korea; 4 Institute of Aging and Metabolic Diseases, College of Medicine, The Catholic University of Korea, Seoul, 06591 South Korea

**Keywords:** Senescence, RNA quality control

## Abstract

HuD, an RNA binding protein, plays a role in the regulation of gene expression in certain types of cells, including neuronal cells and pancreatic β-cells, via RNA metabolism. Its aberrant expression is associated with the pathogenesis of several human diseases. To explore HuD-mediated gene regulation, stable cells expressing short hairpin RNA against HuD were established using mouse neuroblastoma Neuro2a (N2a) cells, which displayed enhanced phenotypic characteristics of cellular senescence. Two approaches, RNA immunoprecipitation (RNA IP)-NanoString profiling and cytokine array, were used to subsequently identify a subset of putative HuD targets that act as senescence-associated secretory phenotype (SASP), including C-C motif ligand 2 (CCL2), CCL20, C-X-C motif chemokine ligand 2 (CXCL2), and interleukin-6 (IL-6). Here, we further demonstrated that HuD regulates the expression of CCL2, a SASP candidate upregulated in cells following HuD knockdown, by binding to the 3′-untranslated region (UTR) of *Ccl2* mRNA. Downregulation of HuD increased the level of CCL2 in N2a cells and the brain tissues of HuD knockout (KO) mice. Exposure to γ-irradiation induced cellular senescence in N2a cells and HuD knockdown facilitated stress-induced cellular senescence. Our results reveal that HuD acts as a novel regulator of CCL2 expression, and its aberrant expression may contribute to cellular senescence by regulating SASP production.

## Introduction

HuD (also known as ELAVL4) is an RNA binding protein belonging to human antigen Hu/ELAVL family. It regulates gene expression at the post-transcriptional level by affecting multiple aspects of RNA metabolism, including stability, translation, alternative splicing, and localization of target mRNAs [1–4]. HuD is predominantly expressed in the brain and plays an important role in brain function; however, it also acts as a pivotal regulator of gene expression in certain types of endocrine cells such as β cells in the islet of pancreas and small cells in the lung (reviewed in [5]). Aberrant expression of HuD is implicated in the pathogenesis of several diseases. For example, several mutations in *HuD* gene are found in patients with Parkinson’s disease [6, 7]. A differential expression of HuD has been reported in Alzheimer’s disease [8, 9], amyotrophic lateral sclerosis [10], schizophrenia [11], pancreatic neuroendocrine tumor [12], and type 2 diabetes [13]. Abnormal phenotypes associated with neural development and synaptic plasticity have also been reported in transgenic or knockout mice against *HuD* [14, 15]. Identification of target mRNAs is essential to understand HuD-mediated gene regulation. Several studies have shown that HuD interacts with various types of RNAs, including mRNA, long non-coding RNA (lncRNA), and circular RNA (circRNA), and regulates dynamic networks of gene expression [16–19]. However, additional efforts in target identification and functional studies may expand our knowledge of the role of HuD in health and disease.

Senescence is originally defined as a stable cell cycle arrest based on the limited capacity for proliferation [20, 21]. Currently, cellular senescence is considered as a response to a wide range of intrinsic and extrinsic stimuli, including genotoxic stress, oxidative stress, mitochondrial dysfunction, oncogene activation, exposure to irradiation, or various reagents [22, 23]. Irreversible cell cycle arrest is a common feature of senescent cells, and is coordinated by several cell cycle regulators; however, certain types of permanently differentiated cells such as neurons, hepatocytes, and adipocytes, also exhibit senescence-like phenotype, which indicates that senescence can occur independent of cell cycle arrest [22].

Senescent cells influence their surrounding environment by generating a senescence-associated secretory phenotype (SASP) [24]. The SASP consists of dynamic and heterogeneous components including cytokines, chemokines, extracellular matrix metalloproteases (MMPs), growth regulators, and angiogenic factors that initiate inflammation, wound healing, and growth responses in nearby cells [25–27]. Recent studies have extensively demonstrated the key role of SASP in various pathophysiological phenomena, including tumorigenesis, angiogenesis, and inflammation, thereby contributing to aging and diseases [23, 28]. Despite the biological significance of SASP, the intracellular signal networks regulating initiation and development of SASP, and the pathological relevance of its aberrant expression are not fully understood.

Herein, we proposed HuD as a novel factor regulating SASP expression in mouse neuroblastoma N2a cells. We found that HuD knockdown increased the levels of senescence-associated β-galactosidase, p16^INK4a^, and reactive oxygen species (ROS), and altered the profile of secreted proteins in the media of N2a cells. In this study, we sought to further identify novel targets of HuD and elucidate HuD-mediated regulatory mechanisms that potentially affect cellular senescence by modifying the microenvironment. Two approaches, RNA immunoprecipitation (RNA IP)-NanoString profiling and cytokine array, were used to subsequently identify a subset of putative HuD targets that function as SASP, including C-C motif ligand 2 (CCL2), CCL20, C-X-C motif chemokine ligand 2 (CXCL2), and interleukin-6 (IL-6). We further demonstrated that HuD regulates the expression of CCL2, a SASP candidate upregulated in HuD knockdown cells, by binding to the 3′-untranslated region (UTR) of *Ccl2* mRNA. Downregulation of HuD not only increased the level of CCL2 in neuroblastoma cells and the brain of HuD knockout (KO) mice, but also increased the susceptibility of cells to stress-induced cellular senescence. Our data reveal that HuD acts as a novel regulator of CCL2 expression, and its aberrant expression may contribute to cellular senescence by regulating SASP production.

## Results

**Fig. 1:**
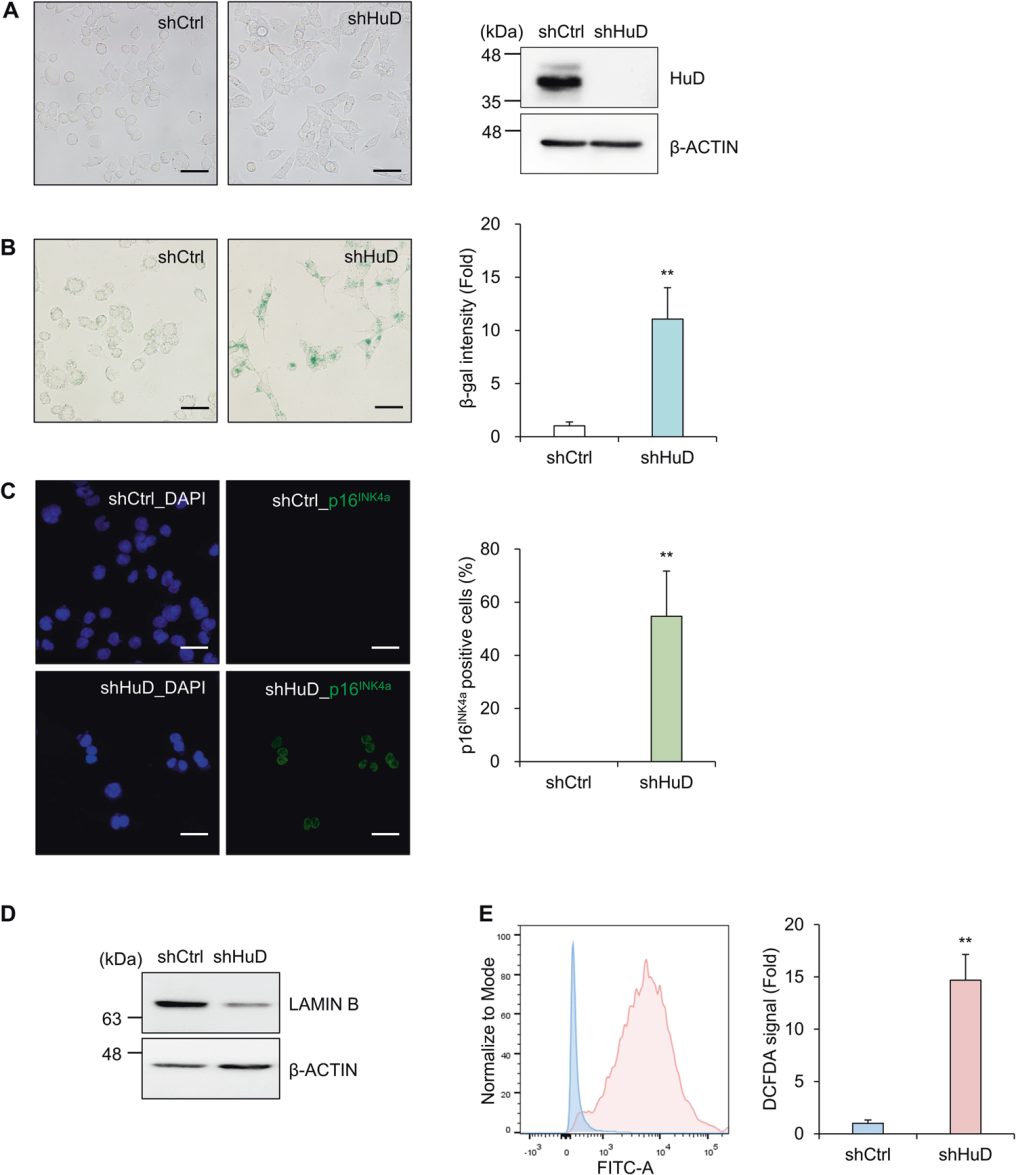
Enhanced cellular senescence mediated via HuD downregulation. **A** Stable cells expressing short hairpin RNA against HuD (shHuD) or control RNA (shCtrl) were established using mouse neuroblastoma Neuro2A (N2a) cells. Downregulation of HuD was assessed via western blotting analysis. β-ACTIN was used as a loading control. **B** Cells were stained with senescence-associated β-galactosidase (SA β-gal). The relative intensity of SA β-gal was assessed via densitometric analysis using Image J. **C** The level of p16^INK4a^ was investigated via immunofluorescence microscopy and quantified by counting the number of p16^INK4a^-expressing cells. Green: p16^INK4a^, blue: nuclei. **D** The level of LAMIN B was assessed via western blotting analysis. β-ACTIN used as a loading control. **E** Cells were incubated with DCFDA, a fluorescent indicator of ROS and cellular ROS levels between N2a_shCtrl and N2a_shHuD cells were determined using flow cytometry. Images are representative, and data indicate the mean ± SEM from three independent analyses. Scale bar, 50 μm. The statistical significance of the data was analyzed via Student’s *t*-test; **p < 0.01.

To identify the target mRNAs of HuD and explore HuD-mediated gene regulatory mechanisms, stable N2a cell lines expressing short hairpin RNA (shRNA) against HuD (shHuD) or control shRNA (shCtrl) were established via continuous selection using puromycin (Fig. 1A). Knockdown of HuD in these cell lines was confirmed by western blotting analysis as shown in Fig. 1A. N2a cells expressing shHuD plasmid (N2a_shHuD) were larger and flatter than control cells (N2a_shCtrl) and expressed higher levels of senescence-associated β-galactosidase (SA β-gal) (Fig. 1B). The number of p16^INK4a^-positive cells increased and the level of LAMIN B decreased in N2a_shHuD cells compared with N2a_shCtrl cells (Fig. 1C, D). In addition, N2a_shHuD cells carried higher levels of ROS than N2a_shCtrl cells (Fig. 1E). These results indicate that N2a_shHuD cells represent senescent phenotypes. Senescent cells are known to secrete various molecules, designated as SASP, and to affect gene expression through autocrine or paracrine pathways [24, 26]. Since HuD is one of pivotal factors in the post-transcriptional control of gene expression, we hypothesized that loss of HuD regulates the expression of SASP factors.

**Fig. 2:**
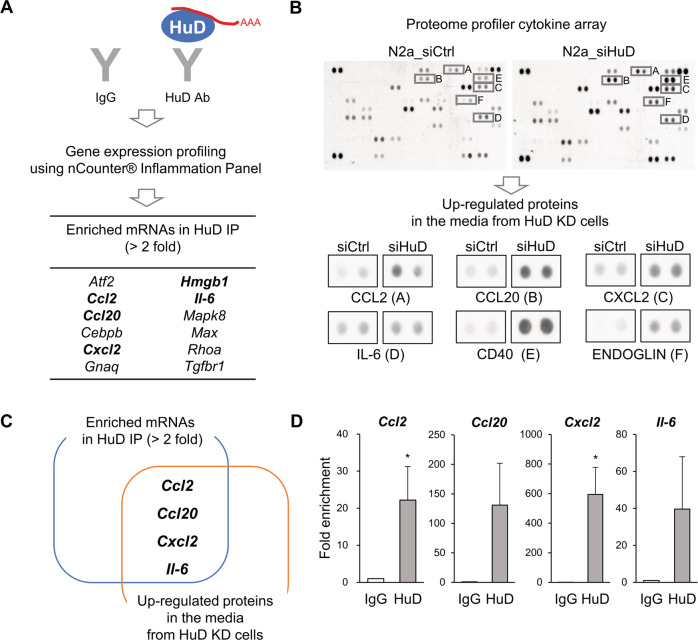
Identification of molecular targets of HuD. **A** The mRNAs that interact with HuD were isolated via RNA immunoprecipitation and analyzed via gene expression profiling using the NanoString nCounter® Inflammation Panel. Enriched mRNAs in HuD IP (>2-fold) compared to control IgG IP are listed in the table and senescence-associated secretory phenotype (SASP) mRNAs are shown in bold. **B** N2a cells were transiently transfected with control siRNA or HuD siRNA and the secretory proteins in the culture media were analyzed by western blotting using the Proteome Profiler Mouse XL cytokine array. The differential expression analysis of secretory proteins revealed six proteins, including CCL2, CCL20, CXCL2, IL-6, CD40, and ENDOGLIN, which were identified as upregulated ones in the media from HuD knockdown cells. **C** Four candidates that not only bind to HuD but also are upregulated by HuD knockdown were determined as putative targets of HuD; *Ccl2*, *Ccl20*, *Cxcl2*, and *Il-6*. **D** Interaction between HuD and its putative targets was validated by RNA IP followed by RT-qPCR using anti-HuD and control IgG antibodies. *Gapdh* mRNA was used for normalization. Data indicate the mean ± SEM from three independent analyses. The statistical significance of the data was analyzed via Student’s *t*-test; *p < 0.05.

In this study, RNA IP-NanoString profiling and cytokine array were used to investigate the role of HuD in the expression of SASP in N2a cells. First, HuD-associating mRNAs were isolated from the RNP complexes containing HuD and analyzed via gene expression profiling using the NanoString nCounter® Inflammation Panel. Enriched mRNAs in HuD IPs were identified as putative target mRNAs of HuD and shown in Fig. 2A. In addition, the differential expression of secretory proteins affected by HuD was determined using the Proteome Profiler Mouse XL Cytokine Array (ARY028). Conditioned media were collected from N2a cells after transfection of either control siRNA or HuD siRNA and the secretory proteins were captured on nitrocellulose membrane and detected with biotinylated detection antibodies and visualized using chemiluminescence reagent according to the manufacturer’s instruction. Upregulated proteins in HuD knockdown cells are shown in Fig. 2B.

These assays revealed four common putative candidates for HuD, including CCL2, CCL20, CXCL2, and IL-6, which are involved in chronic inflammatory response (Fig. 2C). The association between HuD and these target mRNAs was experimentally validated via RNA IP analysis followed by quantitative reverse transcription PCR (RT-qPCR) (Fig. 2D). Taken together, these results suggest the possibility that HuD may play a role in regulating the expression of these secretory proteins known as SASP factors. We further investigated whether HuD regulates the expression of CCL2, one of putative targets, in this study.

**Fig. 3:**
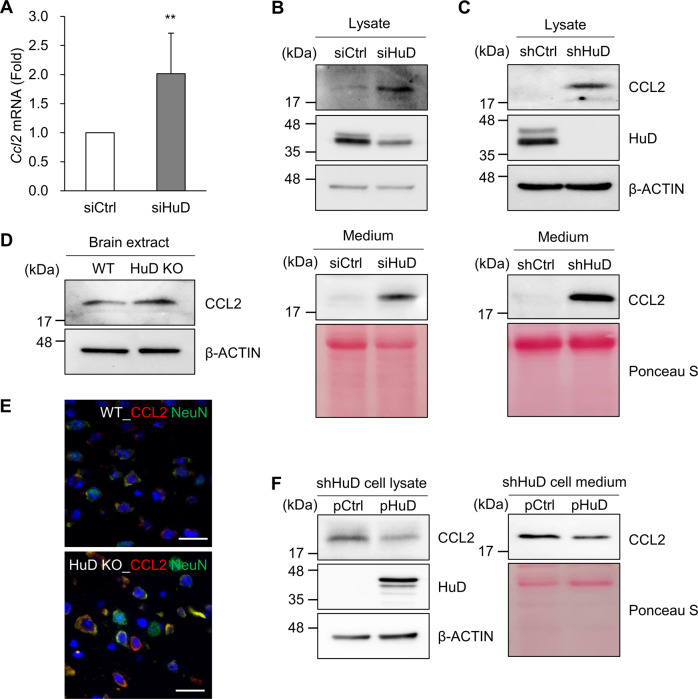
Augmented expression of CCL2 by HuD knockdown. **A** Following the transfection of N2a cells with either control siRNA or HuD siRNA, the expression of *Ccl2* mRNA was determined via RT-qPCR. *Gapdh* mRNA was used for normalization. **B**, **C** The relative levels of CCL2 protein in both lysates (*top*) and culture media (*bottom*) were assessed via western blotting analysis in siRNA transfected cells (**B**) and stable cells (**C**). **D**, **E** Endogenous CCL2 levels in the brain of HuD KO mice and age-matched WT mice were assessed via western blotting analysis (**D**) and immunofluorescence microscopy (**E**). *n* = 3, Red: CCL2, green: NeuN, blue: nuclei. Scale bar, 20 μm. **F** N2a_shHuD cells were transiently transfected with plasmids (pHuD or control plasmid) and CCL2 levels in both lysates and media were determined by western blotting analysis. β-ACTIN blot and Ponceau S staining were used as loading controls. Images are representative, and data indicate the mean ± SEM from three independent experiments. The statistical significance of the data was analyzed via Student’s *t*-test; **p < 0.01.

To determine whether HuD regulates CCL2 expression, the levels of *Ccl2* mRNA and protein were assessed in N2a cells transfected with siRNAs. HuD knockdown moderately increased *Ccl2* mRNA (Fig. 3A) and CCL2 protein in both lysates and media (Fig. 3B). The expression of CCL2 was also augmented in both lysates and media of stable N2a_shHuD cells (Fig. 3C), which indicates that HuD downregulation increases CCL2 expression in N2a cells. An increase of CCL2 by HuD knockdown was also observed in human neuroblastoma SH-SY5Y cells (Supplementary Fig. S1). In addition, the relative CCL2 level was investigated in the brain of HuD knockout (KO) mice or age-matched control brain via western blotting analysis and immunofluorescence microscopy. The results showed that CCL2 expression was increased in the brain of HuD KO mice compared with WT brain (Fig. 3D, E). Fluorescence for CCL2 was predominantly found in NeuN-positive (neuronal) cells and was also observed in some of GFAP-positive (astrocyte), OLIG2-positive (oligodendrocyte), or CD68-positive (macrophage) cells (Supplementary Fig. S2), which suggests that CCL2 increased in several types of cells in the brain of KO mice. To further determine whether HuD regulates CCL2 expression, the HuD level was restored in N2a_shHuD cells via ectopic expression of HuD and the relative expression of CCL2 was investigated. As shown in Fig. 3F, HuD overexpression downregulated CCL2 levels in both lysates and media of N2a_shHuD cells. Together, these results suggest that HuD plays a negative role in the regulation of CCL2 expression.

**Fig. 4:**
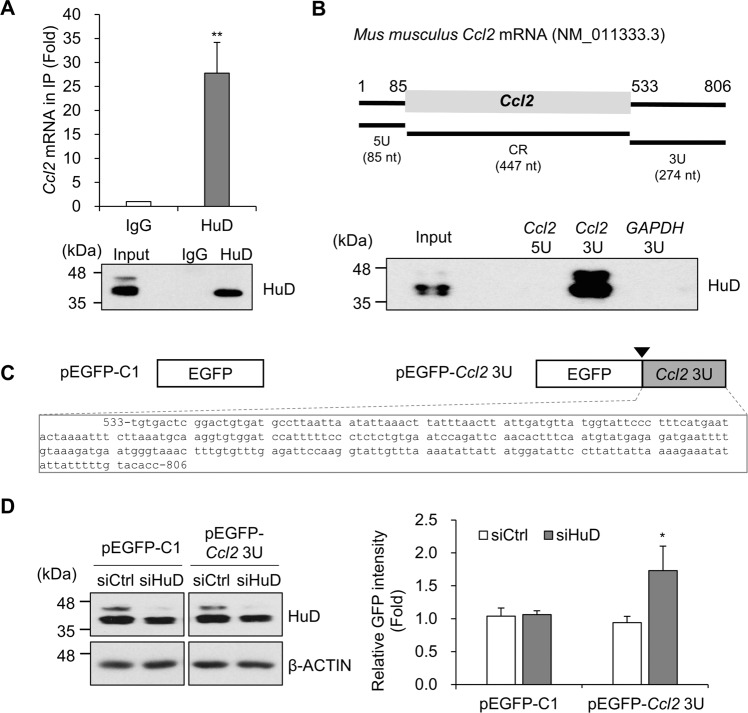
Interaction between HuD and 3′UTR of *Ccl2* mRNA. **A** The interaction between HuD and *Ccl2* mRNA was confirmed by RNA IP followed by RT-qPCR using anti-HuD and control IgG antibodies. *Gapdh* mRNA was used for normalization. **B** *Top:* A schematic of mouse *Ccl2* mRNA (NM_011333.3). The UTRs of *Ccl2* mRNA (5U and 3U) were transcribed in vitro using T7 RNA polymerase and biotin-labeled nucleotides. *Bottom*: The biotinylated transcripts (*Ccl2* 5U, *Ccl2* 3U, and *GAPDH* 3U) were incubated with the lysate from N2a cells. The proteins binding to the transcripts were pulled-down using streptavidin magnetic beads and analyzed via western blotting using HuD antibody. Biotinylated *GAPDH* 3U was used as a negative control. **C** Schematic diagram of EGFP reporters. The reporter plasmid (pEGFP-*Ccl2* 3U) was constructed by inserting the 3′UTR of *Ccl2* mRNA (533–806 nt) into the pEGFP-C1. (▼: stop codon). **D** After sequential transfection with siRNAs and EGFP reporter plasmids, relative fluorescence of EGFP from each sample was measured by fluorescence microscopy and the levels of HuD and β-ACTIN were assessed by western blotting analysis. Images are representative, and data indicate the mean ± SEM from three independent experiments. The statistical significance of the data was analyzed via Student’s *t*-test; *p < 0.05; **p < 0.01.

HuD is an RNA binding protein (RBP) known to interact with target mRNAs and affect their expressions [2, 5]. To understand HuD-mediated regulation of CCL2 expression, the association between HuD and *Ccl2* mRNA was assessed by RNA IP analysis followed by RT-qPCR. *Ccl2* mRNA was enriched in HuD IP (Fig. 4A), which indicates that the HuD-containing RNP complex binds to *Ccl2* mRNA. Since RBPs usually affect the expression of target mRNAs via binding to their UTRs, the association between HuD and UTRs of *Ccl2* mRNA was investigated by the pull-down assay using biotin-labeled probes and the results showed that HuD binds to 3′UTR of *Ccl2* mRNA (Fig. 4B). To further confirm whether HuD regulates CCL2 expression by binding to its 3′UTR, the EGFP reporter plasmid containing the sequence of *Ccl2* 3′UTR (533–806 nt, pEGFP-*Ccl2* 3U) behind the coding region of EGFP was generated (Fig. 4C). The relative EGFP expression was analyzed after transfection of siHuD or control siRNA. As shown in Fig. 4D, HuD knockdown enhanced the fluorescence of EGFP-*Ccl2* 3U reporter plasmid, but not the level of EGFP control. These results suggest that HuD binds to 3′UTR of *Ccl2* mRNA and downregulates its expression.

**Fig. 5:**
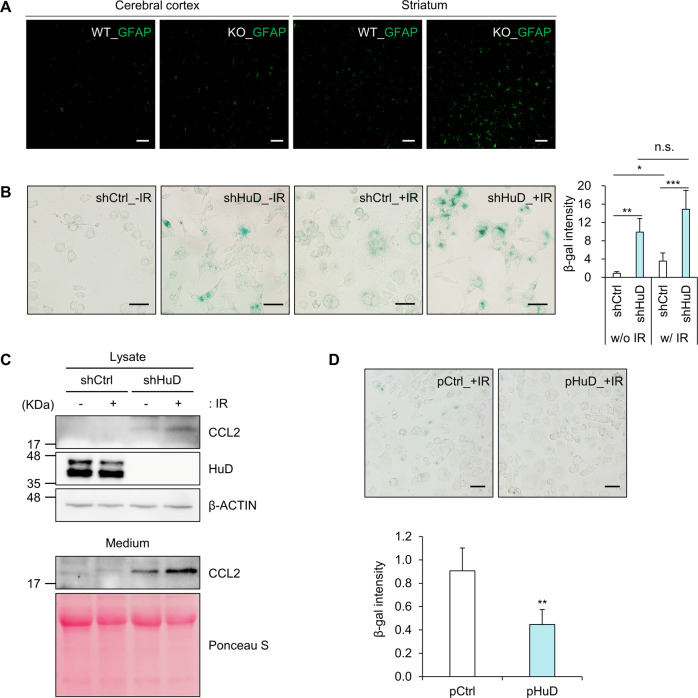
Sensitization of N2a cells via HuD knockdown in response to senescence inducer. **A** Relative level of GFAP, an astrocyte marker, was analyzed in the cerebral cortex and striatum regions in the brain of HuD KO mice and their age-matched control mice by immunofluorescence microscopy. *n* = 3. **B**, **C** After exposure to γ-irradiation (5.5 Gy), the levels of SA β-gal (**B**) and CCL2 expression (**C**) were analyzed by β-gal staining and western blotting analysis. **D** N2a cells were transiently transfected with plasmids and exposed to γ-irradiation. The levels of SA β-gal were assessed by β-gal staining and densitometric analysis using Image J. Scale bar, 50 μm. Images are representative, and data indicate the mean ± SEM derived from three independent experiments. The statistical significance of the data was analyzed via Student’s *t*-test; *p < 0.05; **p < 0.01; ***p < 0.001; n.s., not significant.

CCL2 mediates astrocyte activation and promotes neuroinflammation during brain injury [29, 30]. Since augmented level of CCL2 was observed in the brain of HuD KO mice (Fig. 3D, E), astrocyte activation in the same brain tissues was assessed by immunofluorescence staining using an antibody to glial fibrillary acidic protein (GFAP), a marker for astrocytes. The intensity of GFAP-positive signal was enhanced in both regions of cerebral cortex and striatum in HuD KO mice (Fig. 5A), indicating enhanced astrocyte activation leading to altered brain microenvironment in HuD KO mice. This result suggests that HuD alters the brain microenvironment by regulating the expression of SASP molecules including CCL2 in HuD-expressing cells such as neurons.

The relative level of SA β-gal was elevated in N2a_shHuD cells compared to normal cells (Fig. 1B). This result led us to hypothesize that HuD downregulation promotes cellular senescence in response to extracellular stimuli. To determine whether N2a_shHuD cells are more sensitive to stress-induced cellular senescence, cells are exposed to γ-irradiation (IR), an inducer of senescence, and then relative levels of SA β-gal and CCL2 between N2a_shHuD cells and control cells were determined. In N2a_shHuD group, the intensity of SA β-gal as well as the expression of CCL2 were higher than in the control group after IR exposure (Fig. 5B, C), which suggests that N2a_shHuD cells were more vulnerable to IR. Transient knockdown of HuD also increased the expression of SA β-gal (Supplementary Fig. S3), while HuD overexpression decreased it (Fig. 5D) after exposure to IR. Taken together, these data suggest that HuD acts as a negative regulator of CCL2 expression and downregulation of HuD contributes to facilitating γ-irradiation-induced SA β-gal production.

**Fig. 6:**
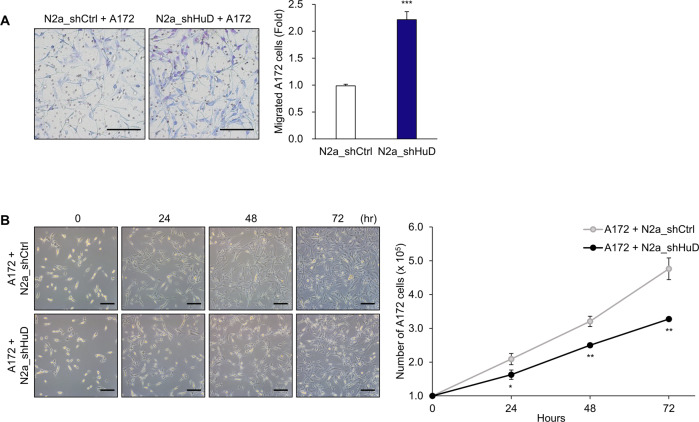
Alterations in migration and growth of A172 cells co-cultured with N2a_shHuD cells. **A** A172 cells (upper chamber) and N2a cells (lower chambers) were co-cultured for 48 h and migration of A172 cells were determined by staining of migrated cells on the transwell membrane. **B** A172 cells (lower chamber) and N2a cells (upper chambers) were co-cultured and the number of A172 cells was analyzed by cell counting at each time point. Images are representative, and data indicate the mean ± SEM derived from three independent experiments. Scale bar, 200 μm. The statistical significance of the data was analyzed via Student’s *t*-test; *p < 0.05; **p < 0.01; ***p < 0.001.

To further investigate whether HuD-mediated CCL2 regulation affects to neighbor cells via paracrine effects, A172 cells were co-cultured with N2a cells and the migration and growth of A172 cells were assessed by the transwell migration assay and cell counting. As shown in Fig. 6, co-culture with N2a_shHuD cells promoted migration of A172 cells (Fig. 6A) and decreased their growth (Fig. 6B) compared to co-culture with N2a_shCtrl cells. These data suggest that HuD has a potential to affect the migration and growth of nearby cells.

## Discussion

HuD is an RBP belonging to Hu antigen family and acts as a pivotal regulator of gene expression at the post-transcriptional level [2, 5]. Differential expression of HuD protein or mutations involving *HuD* gene is implicated in the pathogenesis of several diseases such as Alzheimer’s disease, Parkinson’s disease, cancer, and diabetes [6, 8, 9, 12, 31]. Although HuD has been recognized as an essential factor regulating RNA metabolism, the detailed mechanisms of its expression, molecular targets, and disease relevance remain to be further elucidated. In this study, we established stable N2a cells expressing shRNA against HuD to explore HuD-mediated gene regulation and observed enhanced levels of SA β-gal and ROS in HuD knockdown cells. We identified an SASP molecule, CCL2, as a novel target of HuD and demonstrated that HuD negatively regulated CCL2 expression by binding to its 3′UTR. HuD knockdown increased CCL2 expression and the level of SA β-gal in response to irradiation. In addition, we showed HuD-mediated CCL2 regulation affects the migration and growth of A172 cells via paracrine effects. These results suggest that HuD regulates the expression of SASP molecules, including CCL2, and downregulation of HuD sensitizes the cells to extracellular stimuli, thereby leading to cellular senescence.

Several phenotypic characteristics of senescence were observed, including accumulation of SA β-gal and p16^INK4a^, increased production of ROS, and downregulation of LAMIN B, in N2a cells expressing shRNA against HuD (Fig. 1). In addition, we showed that HuD altered the expression of several secreted proteins such as CCL2, CCL20, IL-6, and CXCL2, suggesting a novel role of HuD in regulating the SASP expression. These results suggest the possibility that HuD not only regulates intracellular gene expression but also mediates cell-to-cell communication. Although downregulation of HuD has been shown in senescent astrocytes [32] using the SeneQuest (http://Senequest.net) provided by the International Cell Senescence Association (ICSA) [23], HuD expression during cellular senescence remains to be elucidated. We observed no significant changes in HuD level after short-term exposure to irradiation (data not shown). However, a follow-up study investigating the changes in HuD expression in response to senescence inducers may provide an important clue to understand the HuD-mediated cell-to-cell crosstalk during cellular senescence.

We previously reported that HuD knockdown induces mitochondrial dysfunction (enhanced mitochondrial fission, reductions in ATP synthesis, mitochondrial membrane potential, and oxygen consumption rate) by regulating *Mfn2* mRNA translation [31]. In addition, several recent articles demonstrate that mitochondrial dysfunction increases SASP production and induces chronic inflammation and/or cellular senescence [33–36]. These suggest that mitochondrial dysfunction is one of important factors facilitating cellular senescence. However, activation of DNA damage response, increased intracellular ROS level, and enhanced oxidative stress response observed in N2a_shHuD cells (data not shown) may act as the positive feedback mechanism in accelerating cellular senescence. Since it is not easy to completely define the role of HuD in the causal relationship between cellular senescence and SASP generation, in this study, we tried to focus on the HuD-mediated SASP regulation. Further studies may be enabled to elucidate a fine mechanism of HuD in the regulation of cellular senescence and age-related diseases.

CCL2 is a proinflammatory chemokine that facilitates the accumulation of immune cells at inflammatory sites [37]. The level of CCL2 is upregulated in several tumors and correlates with poor prognosis of patients with cancers of breast, stomach, and liver [38–40]. CCL2-overexpressing mice exhibit metabolic dysregulation and premature death with accelerating aging [41, 42]. In addition, CCL2 promotes the activation of astrocytes and microglia in the brain [29, 30]. Therefore, understanding of the detailed mechanism of CCL2 expression is important for the development of therapeutic strategies targeting inflammatory response. CCL2 expression is largely controlled via the transcription factor nuclear factor κ B (NFκB) signaling; however, other factors, such as p53 and forkhead box K1 (FOXK1), also regulate the transcription of *Ccl2* [43–45]. Several microRNAs, including miR-33, miR-124, miR-206, and miR-374, negatively regulate CCL2 expression [46–49], while HuR positively regulates it in response to tumor necrosis factor α (TNFα) [50]. Here, we demonstrated that HuD knockdown upregulated CCL2 expression in both mouse neuroblastoma N2a cells and human neuroblastoma SH-SY5Y cells, suggesting a potential role of HuD as a negative regulator of CCL2 expression (Figs. 3 and S1). Although we observed a moderate, but significant, increase in *Ccl2* mRNA level via HuD knockdown (Fig. 3A), the detailed mechanism underlying the decreased expression of CCL2 mediated via HuD needs to be further elucidated.

Several RBPs, including HuD, AU-rich element protein 1 (AUF1), tristetraprolin (TTP), T cell intracellular antigen 1 (TIA1), WIG1, and CUG-binding protein 1 (CUGBP1), are implicated in the regulation of cellular senescence (reviewed in [51]). Loss of HuR has been reported to promote cellular senescence in several cell types, suggesting that HuR plays a suppressive role in senescence [52–56]. Cheng et al. [57] recently reported the roles of HuB and HuD in the regulation of telomerase activity. Interestingly, downregulation of HuB and HuD together increased cell growth and delayed cellular senescence of human neuroblastoma SH-SY5Y cells. Here, we demonstrated that knockdown of HuD promoted cellular senescence and SASP expression in mouse neuroblastoma N2a cells. We did not investigate whether telomerase activity was altered by HuD downregulation or whether HuD regulates SASP expression cooperatively or competitively with other Hu family proteins in our system. Therefore, it is difficult to determine the cause of this difference in senescent phenotypes after HuD regulation. Additional experiments are required to fully understand the regulatory mechanisms of gene expression by Hu family of proteins among different cell types or species.

In conclusion, we propose a novel function of HuD in the regulation of SASP expression. We demonstrated that downregulation of HuD increased the level of SASPs, such as CCL2, CCL20, CXCL2, and IL-6, and sensitized the cells to senescence inducers, thereby promoting cellular senescence. Our results suggest that HuD acts as a novel regulator of CCL2 expression, and its aberrant expression induces cellular senescence by altering SASP production. Further studies investigating the relative level of HuD in response to senescence inducers altered cell-to-cell communication mediated by SASPs in *HuD*-deficient models, and their underlying mechanisms may provide additional insight into HuD-mediated gene regulation during cellular senescence or neuroinflammation.

## Materials and methods

### Cell culture, transfection of plasmids and small interfering RNAs

Mouse neuroblastoma Neuro2a (N2a) and human glioblastoma A172 cells were cultured in Dulbecco’s modified Eagle’s medium (DMEM) and RPMI 1640 (Capricorn Scientific, Ebsdorfergrund, Germany) supplemented with 10% fetal bovine serum (FBS) and 1% antibiotics and incubated at 37 °C in the presence of 5% CO_2_. Stable cells expressing shRNAs were established by transfection of shHuD plasmid (Santa Cruz Biotechnology, Inc., Dallas, TX, USA) or control plasmid under puromycin (Invitrogen™, Waltham, MA, USA) selection. Enhanced green fluorescent protein (EGFP) reporter was prepared by cloning the 3′UTR sequence of *Ccl2* mRNA (533–806, 274 nt) into the pEGFP-C1 (BD Bioscience, Franklin Lakes, NJ, USA) vector. HuD overexpression plasmids (pHuD) were received as a gift from Prof. Alessandro Quattrone [18]. Transfection of small interfering RNAs (HuD siRNA (siHuD) and control siRNA (siCtrl)) (Genolution Pharmaceuticals, Inc., Seoul, South Korea) or plasmids were achieved using Lipofectamine™ 2000 (Invitrogen™) according to the manufacturer’s instructions.

### RNA analysis

Total RNA was isolated from whole cells using RNAiso Plus (Takara Bio, Inc., Shiga, Japan) and cDNA was synthesized by reverse transcription using ReverTra® Ace qPCR RT kit (Toyobo Co., Ltd, Osaka, Japan). The level of transcripts was determined via quantitative PCR (qPCR) using the SensiFAST™ SYBR Hi-ROX kit (Meridian Bioscience, Inc., Cincinnati, OH, USA), gene-specific primers (Supplementary Table S1), and StepOnePlus™ Real-Time PCR System (Applied Biosystems™, Waltham, MA, USA). Data were processed using the ΔΔCT method for comparison between control and experimental groups. *Gapdh* mRNA was used for normalization.

For RNA immunoprecipitation, ribonucleoprotein (RNP) complexes were immunoprecipitated from the cell lysates using Protein A bead (Invitrogen™) incubated with anti-HuD or control IgG antibody (Santa Cruz Biotechnology, Inc.) [58]. The immunoprecipitated RNP complexes were sequentially incubated with DNase I and proteinase K. The isolated RNAs from the complexes were used for further analysis including RT-qPCR and gene profiling using the nCounter® Analysis Systems (NanoString Technologies, Inc., Seattle, WA, USA). Gene expression profiling in the complexes was accomplished by using the nCounter® Inflammation Panel. Data were analyzed using nSolver software according to the instruction.

### Biotin pull down assay

To synthesize biotinylated transcripts, DNA fragments corresponding to the 5′UTR and 3′UTR of *Ccl2* mRNA (NM_011333.3) were generated using forward primers containing T7 RNA polymerase binding sequence (5′-CCAAGCTTCTAATACGACTCACTATAGGGAGA-3′). After purification of the PCR products, biotinylated transcripts were synthesized using the MaxiScript T7 kit (Invitrogen™) and biotin-CTP (Enzo Life Sciences, Inc., Farmingdale, NY, USA). Cell lysates were incubated with the purified biotinylated transcripts for 30 min at room temperature. The RNA–protein complexes were isolated using streptavidin-coupled Dynabeads (Invitrogen™). Proteins were isolated from the complex and subjected to western blotting analysis using HuD antibody [58]. 3′UTR of *Gapdh* mRNA was used as a negative control for the binding assay.

### Western blotting analysis

Whole-cell lysates were prepared using RIPA buffer (Biosesang, Inc., Seongnam, South Korea) containing protease inhibitor cocktail (Roche, Basel, Switzerland). The samples were separated by SDS polyacrylamide gel electrophoresis (SDS-PAGE), transferred onto polyvinylidene difluoride (PVDF) membranes (Millipore, Burlington, MA, USA), incubated with primary antibodies including CCL2 (Abcam Plc., Cambridge, UK), HuD, LAMIN B, GFP (Santa Cruz Biotechnology, Inc.), and β-ACTIN (Genetex, Inc., Irvine, CA, USA) at 4 °C overnight, and further incubated with horseradish peroxidase (HRP)-conjugated secondary antibodies (Sigma-Aldrich, Burlington, MA, USA). Chemiluminescence was detected with the Clarity Western ECL Substrate (Bio-Rad, Inc., Hercules, CA, USA) using the ChemiDoc Imaging Systems (Bio-Rad, Inc.).

### Cytokine array

Conditioned media collected from the cell culture were centrifuged to remove cell debris and used for the analysis of secretory proteins. Cytokine array was performed using the Proteome Profiler Mouse XL Cytokine Array Kit (Cat. No. ARY028, R&D Systems, Inc., Minneapolis, MN, USA) according to the manufacturer’s instructions. In brief, after transfection of N2a cells with siCtrl or siHuD, the conditioned media from each cell were collected to 50 mL tube and concentrated by centrifugation using Amicon® Ultracentrifugal filter (Millipore). The concentrated media were incubated the nitrocellulose membrane containing 111 different anti-mouse cytokine antibodies from the Mouse XL Cytokine Array kit. Captured proteins on the membranes were further incubated with detection antibodies and visualized using chemiluminescent detection reagents.

### Measurement of reactive oxygen species

The intracellular ROS level was determined using a general oxidative stress indicator, CM-H2DCFDA (DCFDA) (Invitrogen™). Cells were incubated with DCFDA reagent at 37 °C for 30 min and washed with Hank’s balanced salt solution (HBSS) (Gibco™, Waltham, MA, USA). The fluorescence signal was analyzed by detecting the DCF intensity using the FACSCanto™ II Flow Cytometry System (BD Bioscience).

### Cell staining and fluorescence microscopy

Cells were fixed with 4% FA solution for immunofluorescence microscopy and brain tissues embedded with paraffin (50 weeks, female mice) were deparaffinized for fluorescence immunohistochemistry. After permeabilization with Triton X-100, cells were sequentially incubated with blocking solution and primary antibodies raised against p16^INK4a^ (Santa Cruz Biotechnology, Inc.), CCL2 (Abcam Plc.), NeuN (Abcam Plc.) and GFAP (Sigma-Aldrich) at 4 °C overnight, and further incubated with secondary antibodies conjugated with Alexa Flour® 488 or Alexa Flour® 555 (Abcam Plc.). DAPI (4′,6-diamidino-2-phenylindole) solution (Invitrogen™) was used to stain the nuclei. Fluorescence signals were observed and imaged using the ZEISS Axio Imager M1 microscope (Carl Zeiss, Oberkochen, Germany).

For detection of senescence-associated β-galactosidase (SA β-gal), cells were fixed with 4% FA and incubated with the SA β-gal solution containing 40 mM citric acid/sodium phosphate (pH 6.0), 150 mM NaCl, 2 mM MgCl_2_, 5 mM potassium ferricyanide and potassium ferrocyanide, and 1 mg/mL X-gal (BEAMS Biotechnology, Seongnam, South Korea) at 37 °C for 16 h in the dark. After washing with PBS, stained cells were observed under the IX70w microscope (Olympus Corp., Tokyo, Japan). The intensity of β-gal was derived by dividing the value of β-gal expression quantified using ImageJ software [59], by the total number of cells.

### Transwell migration assay and cell counting

Transwell migration assay was performed using a Falcon® Permeable Support for 24-well Plate with 8.0 μm Transparent Membrane (Corning Inc., NY, USA). N2a shCtrl and shHuD cells were cultured in a lower chamber, while A172 cells were added in an upper chamber. After incubating A172 cells with RPMI media containing 1% FBS for 48 h, the membrane was stained with a Diff Quit kit (Sysmex Asia Pacific Pte Ltd, Singapore) and observed under a ZEISS Axio Imager A1 microscope (Carl Zeiss). For cell counting assay, A172 cells were seeded in the lower chamber of a Falcon® 6-well TC-treated Polystyrene Permeable Support Companion Plate and N2a cells were added in upper chambers. The number of A172 cells were determined by cell counting with a hemocytometer under the Leica DM IL LED microscope (Leica Microsystems Ltd, Wetzlar, Germany).

### Statistical analysis

Data were expressed as mean ± SEM of three independent experiments. The statistical significance of the data was analyzed via Student’s *t*-test (*p < 0.05; **p < 0.01; ***p < 0.001).

### Reporting summary

Further information on research design is available in the Nature Research Reporting Summary linked to this article.

## Supplementary information

Supplementary materials

Reporting summary

## Data Availability

All data needed to evaluate the conclusions in the paper are present in the paper. Additional data may be available from the corresponding author on reasonable request.
