# Supplementary material for: Loss of RNA binding protein HuD facilitates the production of the senescence-associated secretory phenotype
Source: Cell Death Dis. 2022 Apr 11;13(4):329. doi: 10.1038/s41419-022-04792-y (PMC9001635; doi:10.1038/s41419-022-04792-y)
Supplement: Supplementary file 1 — Supplementary materials [file 41419_2022_4792_MOESM1_ESM.docx]

**Loss of RNA binding protein HuD facilitates the production of the senescence-associated secretory phenotype.**

​Seungyeon Ryu^1,2^, Myeongwoo Jung^1,2^, Chongtae Kim^2,3^, Hoin Kang^1^, Sukyoung Han^1,2^, Seongho Cha^1,2^, Seung Min Jeong ^1, 2, 4^, Eun Kyung Lee^1, 2, 4^ *

^1^ Department of Biomedicine & Health Sciences, ^2^ Department of Biochemistry, ^3^ Catholic Institute for Visual Science, ^4^ Institute of Aging and Metabolic Diseases, College of Medicine, The Catholic University of Korea, Seoul 06591, South Korea

* Correspondence should be addressed to Eun Kyung Lee; [leeek@catholic.ac.kr](mailto:leeek@catholic.ac.kr)

**Materials and Methods**

**Cell culture and transfection of small interfering RNAs**

Human neuroblastoma SH-SY5Y cells were cultured in Dulbecco’s modified Eagle’s medium (DMEM) (Capricorn Scientific) supplemented with 10% fetal bovine serum and 1% antibiotics and incubated at 37°C in the presence of 5% CO_2_. Transfection of small interfering RNAs was achieved using Lipofectamine™ 2000 (Invitrogen™).

**Immunofluorescence microscopy**

Brain tissues were deparaffinized, permeabilized, and incubated with primary antibodies raised against CCL2 (Abcam Plc.), NeuN (Abcam Plc.), OLIG2 (GeneTex, Inc.), CD68 (Abcam Plc.), and GFAP (Sigma-Aldrich) at 4°C overnight, and further incubated with secondary antibodies conjugated with Alexa Flour® 488 or Alexa Flour® 555 (Abcam Plc.). DAPI (4',6-diamidino-2-phenylindole) solution (Invitrogen™) was used to stain the nuclei. Fluorescence signals were observed and imaged using the ZEISS Axio Imager M1 microscope (Carl Zeiss).

**Supplementary Figure S1. Augmented expression of CCL2 by HuD knockdown in SH-SY5Y cells**

**
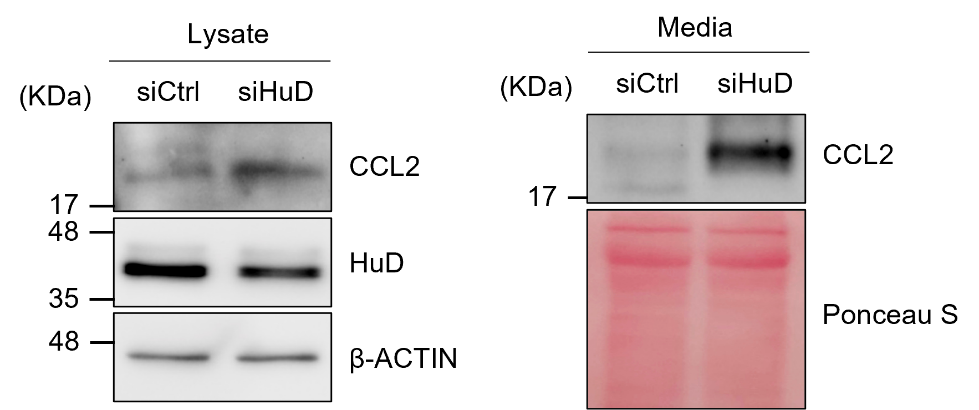
**

Following the transfection of human neuroblastoma SH-SY5Y cells with either control siRNA or HuD siRNA, the expression of CCL2 protein in both lysate (left) and culture medium (right) was assessed via western blotting analysis. β-ACTIN blot and Ponceau S staining were used as loading controls.

**
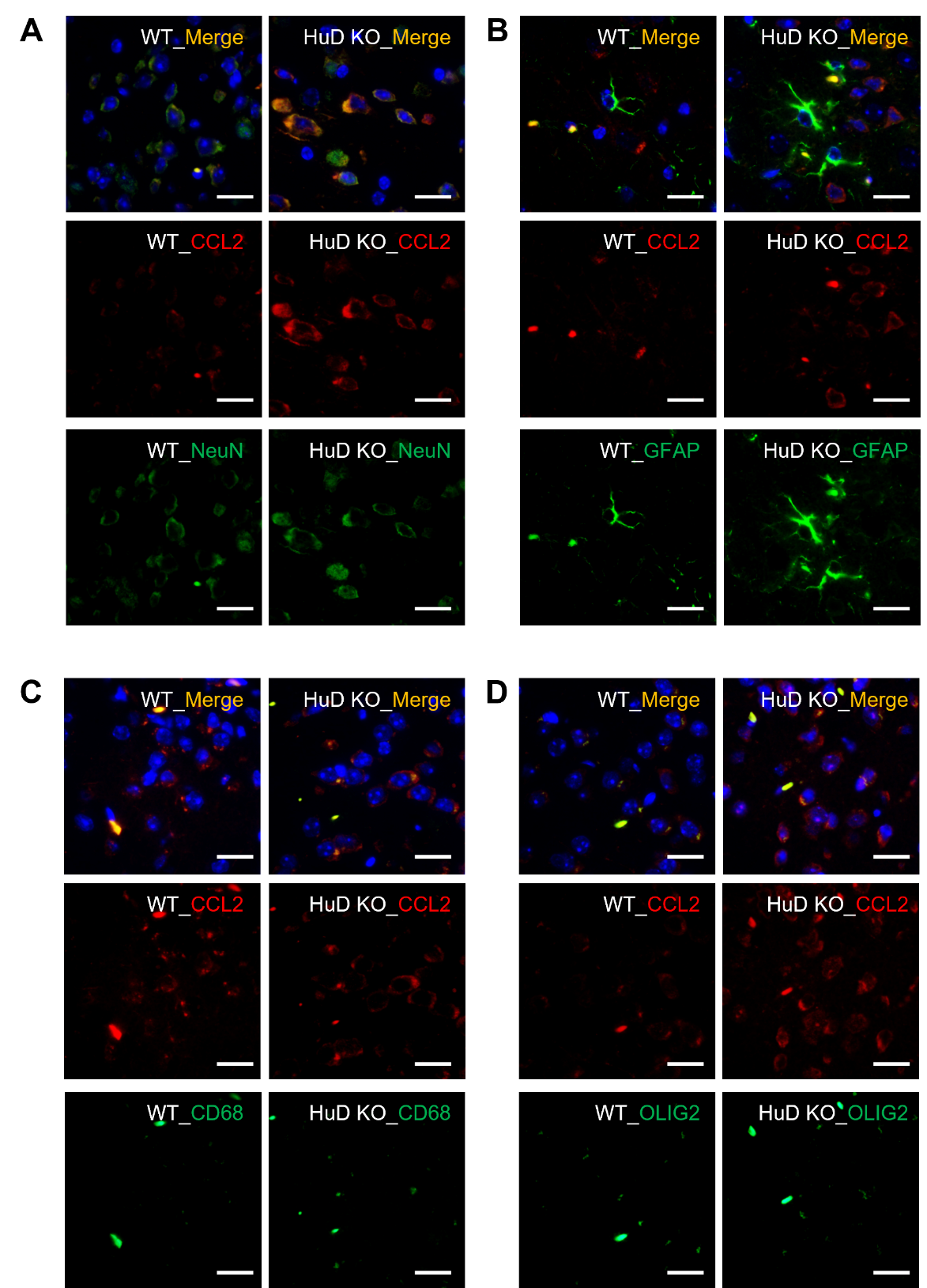
Supplementary Figure S2. Expression of CCL2 in the brain tissues**

Endogenous CCL2 levels in the brain of HuD KO mice and age-matched WT mice were assessed via fluorescence microscope by co-staining with several marker proteins, including NeuN (neuron), GFAP (astrocyte), CD68 (macrophage), and OLIG2 (oligodendrocyte). Red: CCL2, Blue: nuclei, Green: NeuN (A), GFAP (B), CD68 (C), and OLIG2 (D). Scale bar, 20 μm.

**Supplementary Figure S3. Expression of SA β-gal after** **exposure to γ-irradiation**

**
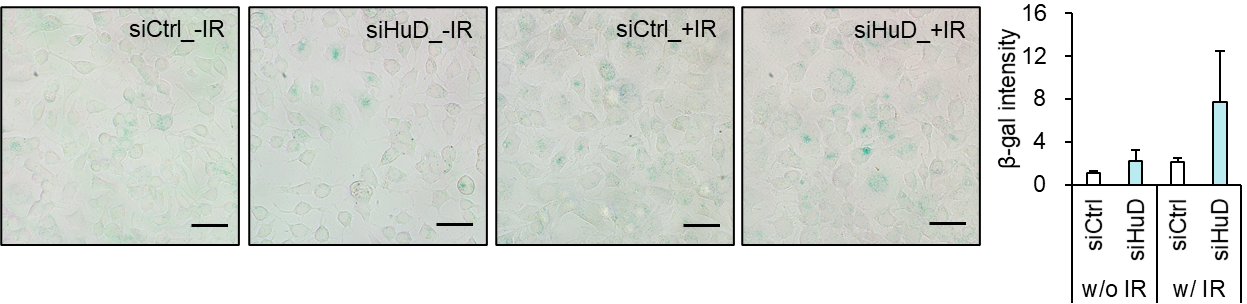
**

N2a cells transfected with either control siRNA or HuD siRNA were exposure to γ-irradiation (5.5 Gy) and the levels of SA β-gal were analyzed by β-gal staining. The intensity of SA β-gal was quantified by densitometric analysis using Image J. Scale bar, 50 μm. The statistical significance of the data was analyzed via Student’s t-test; n.s. (not significant).

**Supplementary table S1. List of primers used in this study**

|  | **Forward primer** | **Reverse primer** |
| --- | --- | --- |
| Mouse *Gapdh* | AGGTCGGTGTGAACGGATTTG | TGTAGACCATGTAGTTGAGGTCA |
| Mouse *HuD* | GCCTCAGGTGTCAAATGGACC | CCATACCCTAAACTCTGTCCTGT |
| Mouse *Ccl2* | TTAAAAACCTGGATCGGAACCAA | GCATTAGCTTCAGATTTACGGGT |
| Mouse *Cxcl2* | AAGTCATAGCCACTCTCAAGGG | GTTAGCCTTGCCTTTGTTCAGTA |
| Mouse *Il6* | TAGTCCTTCCTACCCCAATTTCC | TTGGTCCTTAGCCACTCCTTC |
| Mouse *Ccl20* | ACTGTTGCCTCTCGTACATACA | GAGGAGGTTCACAGCCCTTTT |
| *Ccl2*-5′UTR | CCAAGCTTCTAATACGACTCACTATAGGGAGAGCAGAGAGCCAGACGGGAGGAA | GGTGGTGGAGGAAGAGAGAGCTGG |
| *Ccl2*-3′UTR | CCAAGCTTCTAATACGACTCACTATAGGGAGATAGTGTGACTCGGACTGTGAT | GGTGTACAAAAATAATATATTTC |
| *GAPDH*-3′UTR | CCAAGCTTCTAATACGACTCACTATAGGGAGACCTCAACGACCACTTTGTCA | GGTTGAGCACAGGGTACTTTAT |
| *Ccl2*-3′UTR  (For reporter assay) | GACCAGATCTTAATGTGACTCGGACTGTGATGC | CTGAGGTACCGGTGTACAAAAATAATATATTTC |
|  |  |  |

**Supplementary table S2. List of antibodies used in this study**

| **Antibody** | **Company** | **Catalog number** |
| --- | --- | --- |
| HuD | Santa Cruz Biotechnology, Inc. | sc-28299 |
| β-ACTIN | GeneTex, Inc. | GTX629630 |
| CCL2 | Abcam Plc. | ab25124 |
| LAMIN B | Santa Cruz Biotechnology, Inc. | sc-6216 |
| p16^INK4a^ | Santa Cruz Biotechnology, Inc. | sc-1661 |
| GFP | Santa Cruz Biotechnology, Inc. | sc-9996 |
| GFAP | Sigma-Aldrich | MAB360 |
| NeuN | Abcam Plc. | ab104224 |
| OLIG2 | GeneTex, Inc. | GTX01779 |
| CD68 | Abcam Plc. | ab31630 |
| Mouse IgG (HRP Conjugated) | Sigma-Aldrich | AP124P |
| Rabbit IgG (HRP Conjugated) | Sigma-Aldrich | AP132P |
| Mouse IgG H&L (Alexa Fluor® 488) | Abcam Plc. | ab150113 |
| Rabbit IgG H&L (Alexa Fluor® 555) | Abcam Plc. | ab150074 |
| Normal mouse IgG | Santa Cruz Biotechnology, Inc. | sc-2025 |
|  |  |  |

**Uncropped western blots**

**
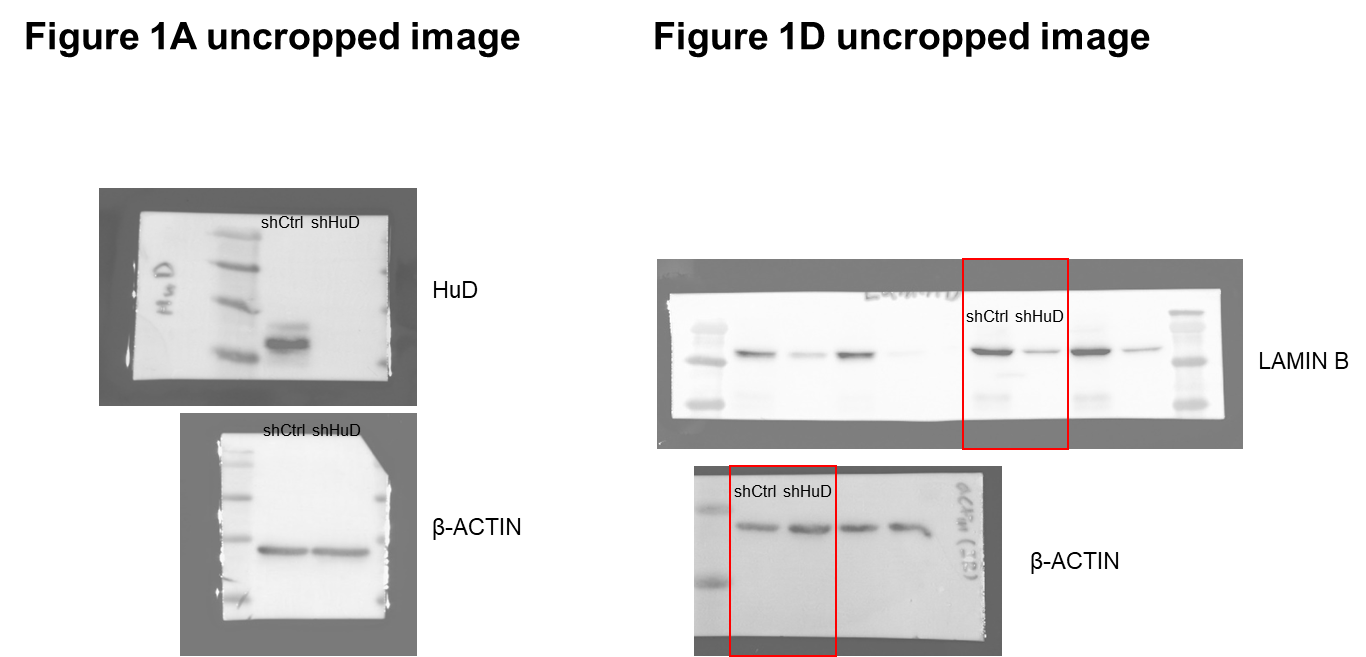
**

**
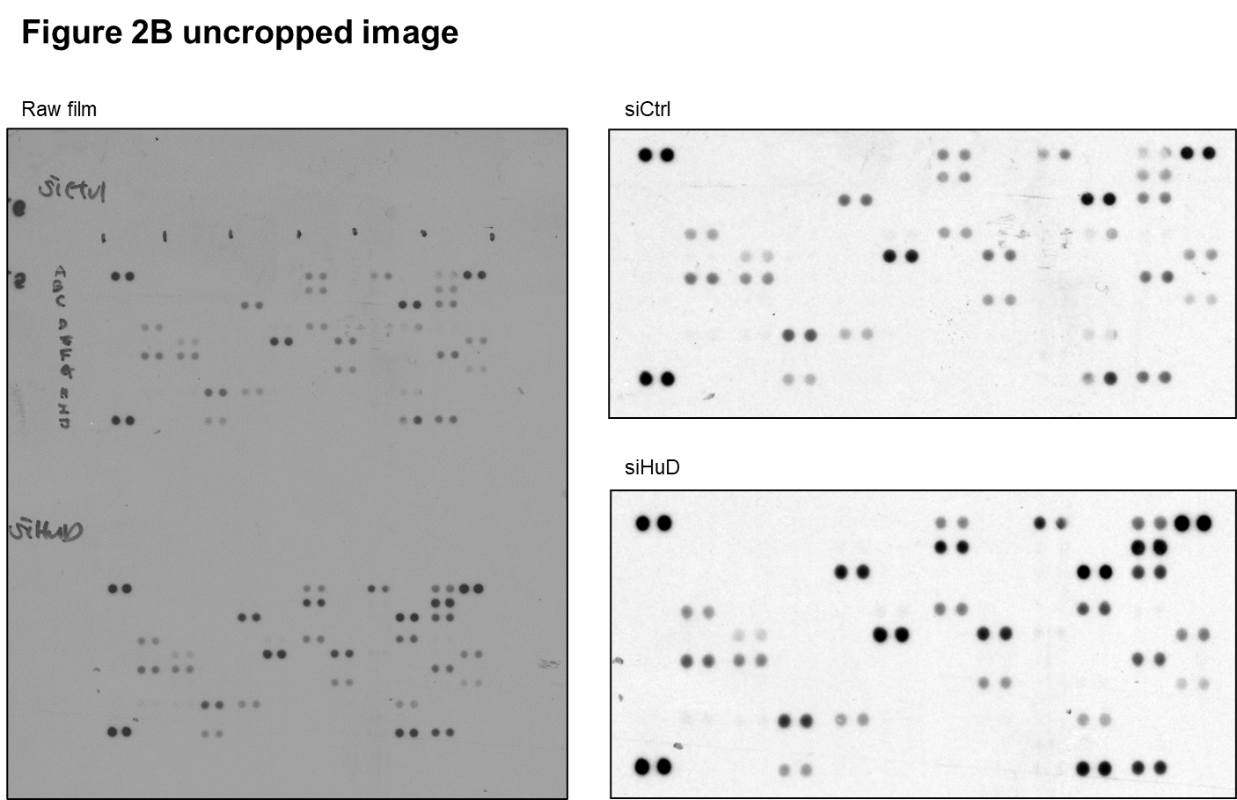
**

**
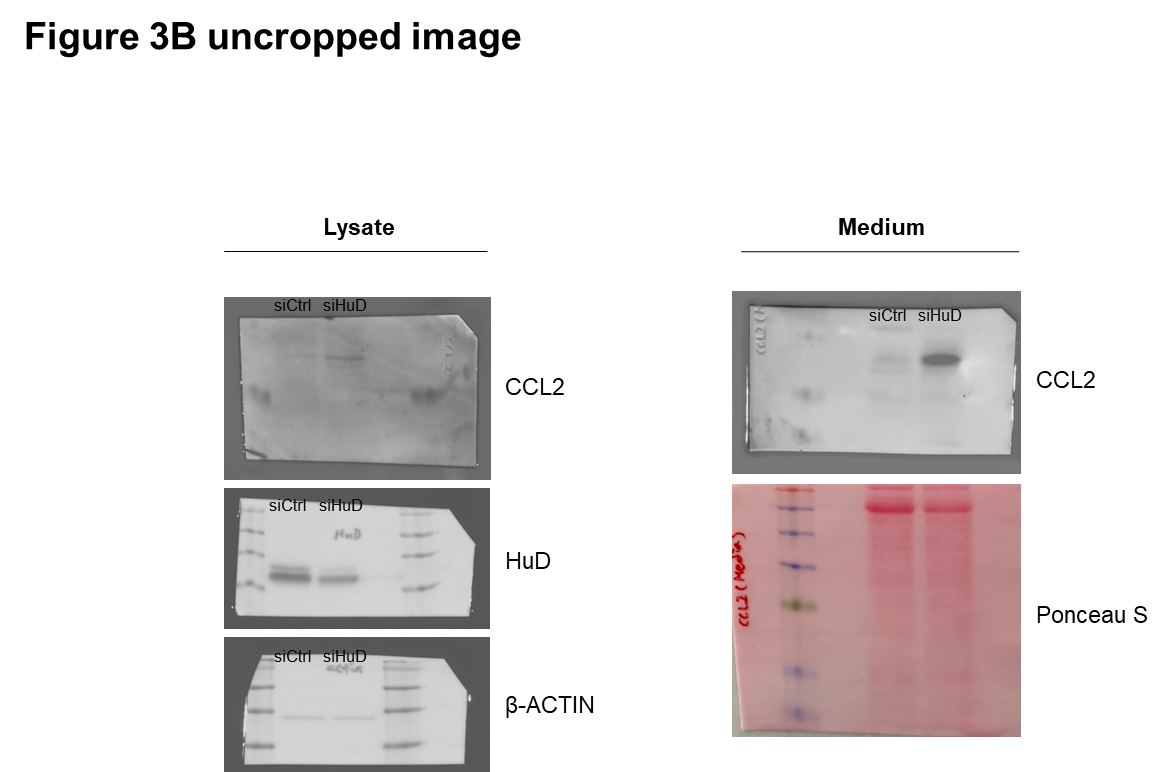
**

**
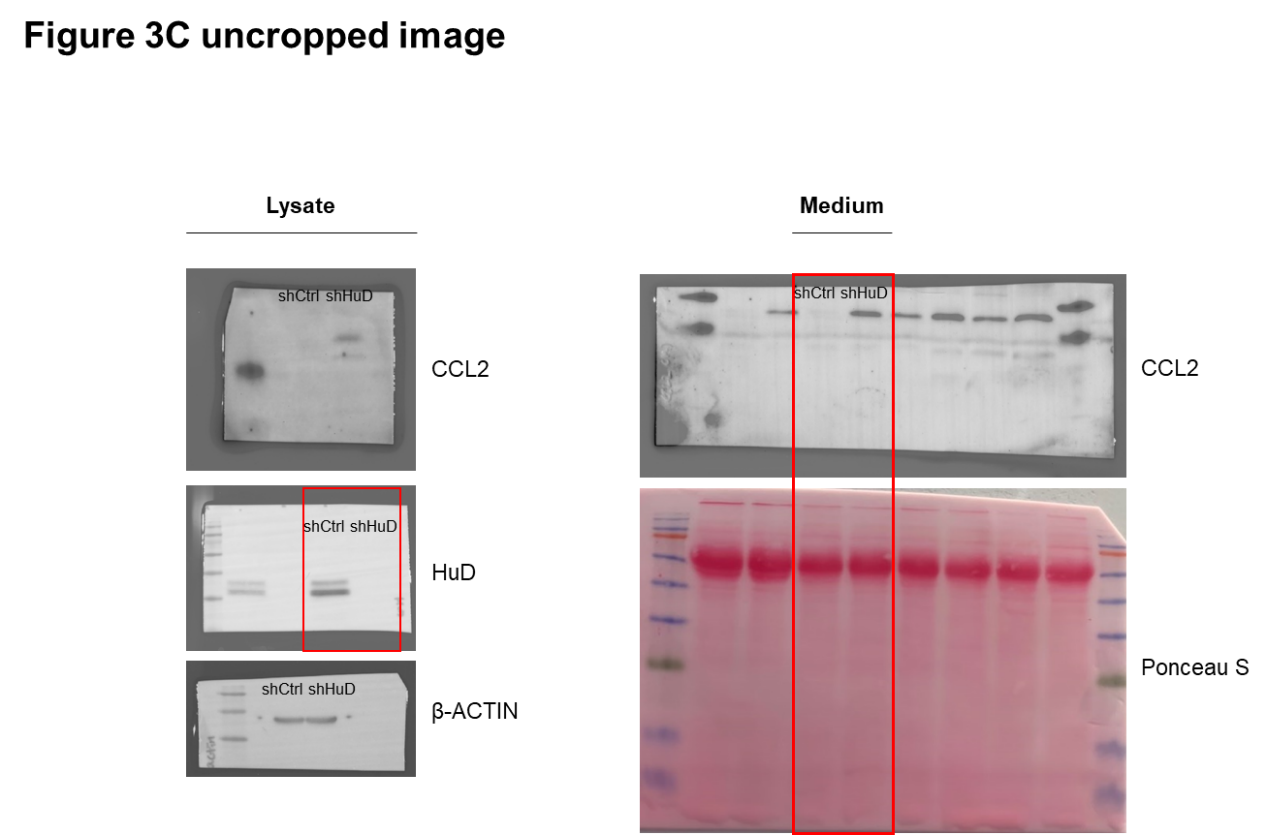
**

**
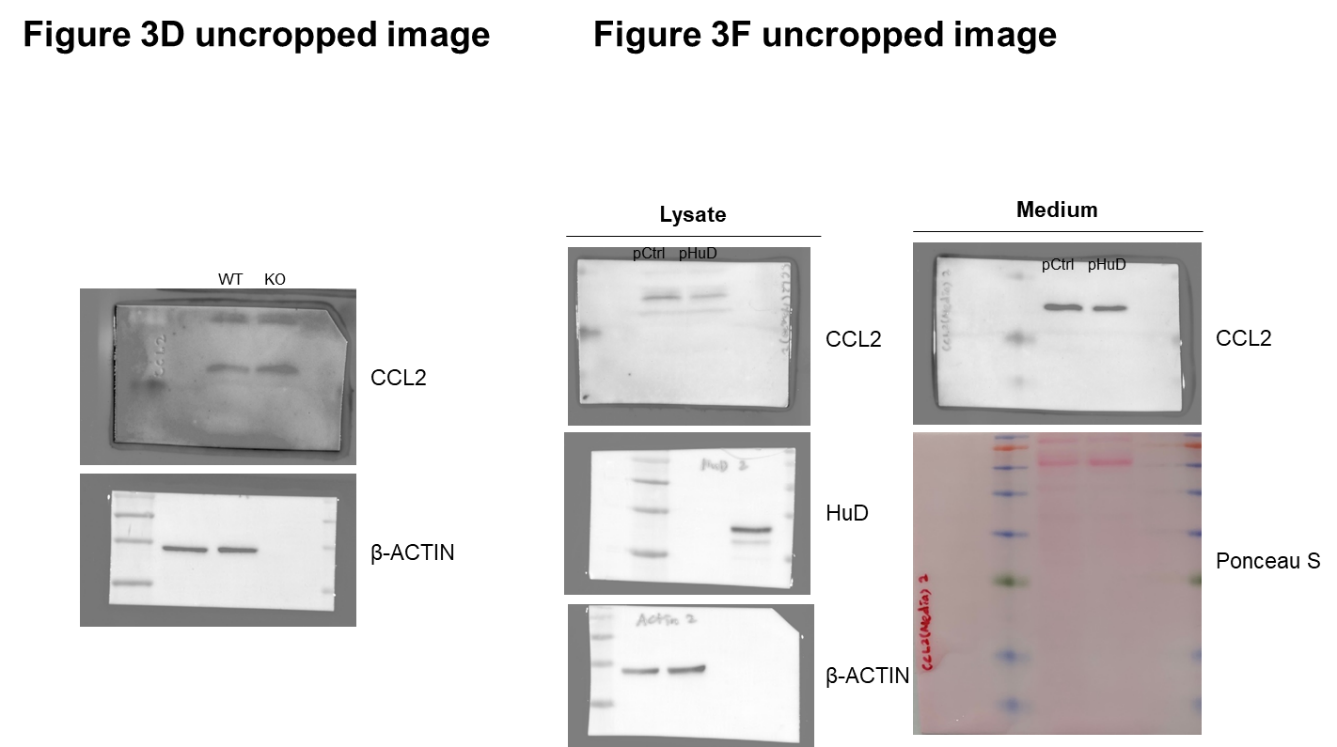
**

**
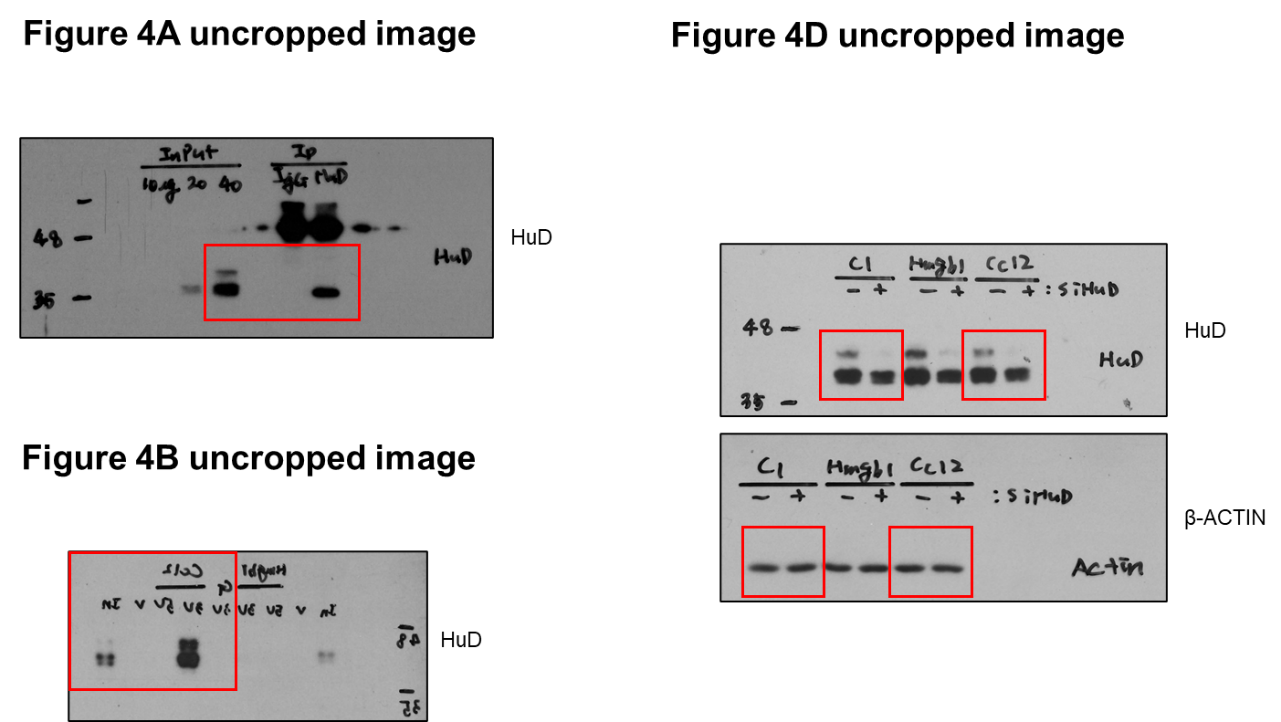
**

**
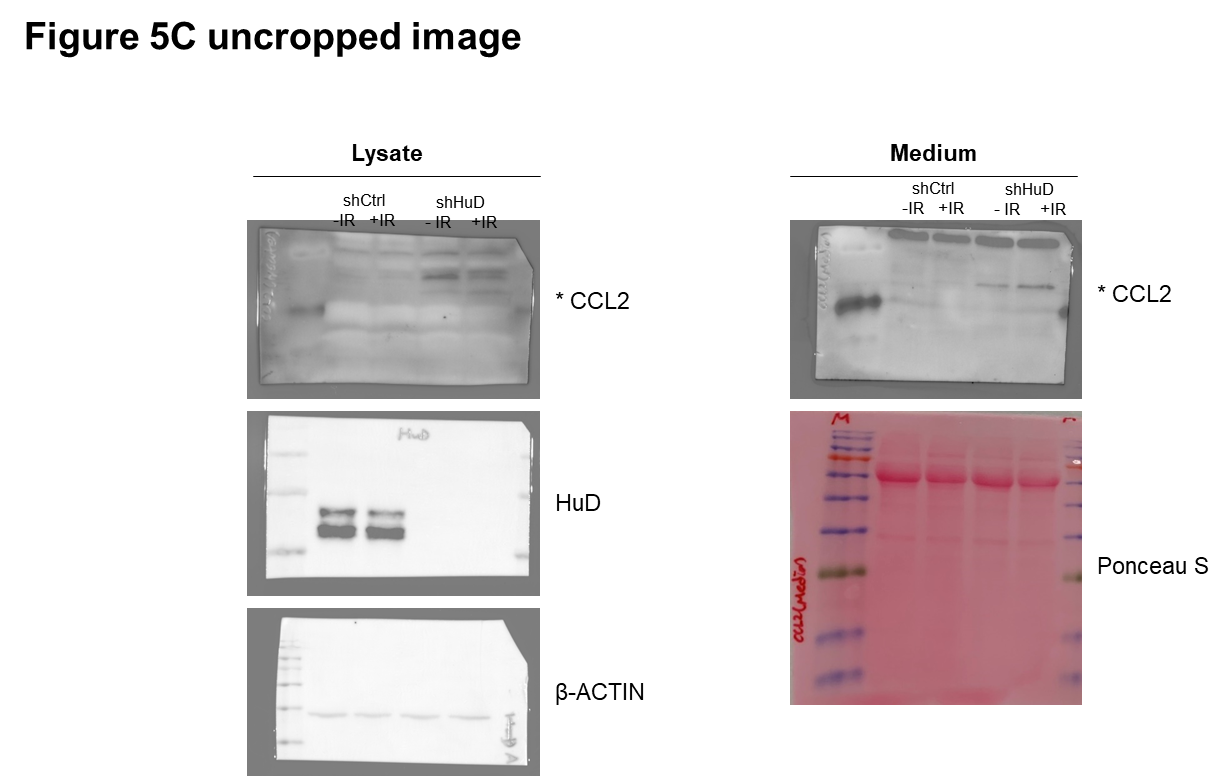
**
